# The effect of catch-up growth in the first year of life on later wheezing phenotypes

**DOI:** 10.1183/13993003.00884-2020

**Published:** 2020-12-24

**Authors:** Sarah J. Kotecha, John Lowe, Raquel Granell, W. John Watkins, A. John Henderson, Sailesh Kotecha

**Affiliations:** 1 Dept of Child Health, Cardiff University School of Medicine, Cardiff, UK; 2 MRC Integrative Epidemiology Unit, Population Health Sciences, Bristol Medical School, University of Bristol, Bristol, UK; 3 Joint first authors

## Abstract

Although wheezing phenotypes have previously been accurately described using well-defined cohorts reporting longitudinal wheezing, early-life factors which lead to development of each wheezing phenotype remain uncertain [1, 2]. Birth weight and catch-up growth affect later respiratory outcomes [3], but the influence of weight gain on specific wheezing phenotypes in term-born children has not been described. Rapid weight gain in early-life is associated with increased rates of childhood wheeze and lower lung function [4, 5]. In one meta-analysis rapid infant weight gain was linked to pre-school wheeze and school-aged asthma; and to increased childhood respiratory symptoms in another meta-analysis [6, 7]. Effect of weight gain in early-life in term-born children on wheezing in early-life is less well reported [8]. In contrast, rapid increase in body mass index in infancy is associated with increased risk of asthma at school-age in preterm-born infants [9].

## Abstract 2

**This study shows that catch-up growth in infancy is associated with increased early wheeze in childhood, so care is needed to not to excessively feed in early infancy** https://bit.ly/2YPxtBw

*To the Editor*:

Although wheezing phenotypes have previously been accurately described using well-defined cohorts reporting longitudinal wheezing, early-life factors which lead to development of each wheezing phenotype remain uncertain [1, 2]. Birth weight and catch-up growth affect later respiratory outcomes [3], but the influence of weight gain on specific wheezing phenotypes in term-born children has not been described. Rapid weight gain in early-life is associated with increased rates of childhood wheeze and lower lung function [4, 5]. In one meta-analysis rapid infant weight gain was linked to pre-school wheeze and school-aged asthma; and to increased childhood respiratory symptoms in another meta-analysis [6, 7]. Effect of weight gain in early-life in term-born children on wheezing in early-life is less well reported [8]. In contrast, rapid increase in body mass index in infancy is associated with increased risk of asthma at school-age in preterm-born infants [9].

Since it is unclear if rapid weight gain in early-life affects any specific wheezing phenotype in term-born children, we investigated if catch-up growth in the first year of life was associated with any specific wheezing phenotypes.

We confined our analyses to term-born (≥37 weeks' gestation), singleton, Caucasian children from the Millennium Cohort Study (MCS), who were born in the UK between 2000 and 2002 [10, 11]. Data on respiratory symptoms (including “wheeze-ever” and “recent wheeze”, defined as parental reporting of wheezing or whistling in the chest in the past 12 months) were collected at 3, 5, 7 and 11 years of age at face-to-face interviews by trained interviewers. The longitudinal wheezing phenotypes were derived using data-driven methods (latent class analysis) using Latent GOLD, v5.1 (Statistical Innovations, Arlington, MA, USA), as previously described [12]. For each child, we assigned the most-likely phenotype (using the highest posterior probability). We conducted sensitivity analyses using weighted phenotypic breakdown thereby splitting each subject proportionately between phenotypes.

Birth weight and weight z-scores adjusted for gestational age and gender were calculated using the LMS Growth program (Medical Research Council, UK) using the British 1990 reference values [13]. Catch-up growth was defined as a change in z-score of >0.67 between birth weight and available weight nearest to 9 months (range 6–12 months). Intrauterine growth restriction (IUGR) and control group were defined as <10^th^ and 20–80^th^ centile for birth weight, respectively, corrected for gender and gestational age [3]. Antenatal smoking was any maternal antenatal smoking and exposure to post-natal smoking was positive if exposure occurred at any time-point in the same room as the child. Caesarean section (CS) was emergency or elective. Social economic status (SES) was defined from the five classes of National Statistics socioeconomic classification based on the last known maternal occupation. Breastfeeding was breast milk intake for any period of time. Childcare use was informal, *e.g.* family members, or formal, *e.g.* childminder or nursery.

Multinomial logistic regression was performed and relative risk ratios and corresponding 95% confidence intervals for associations between catch-up growth in the first year and wheezing phenotypes (3 to 11 years) are reported. The “No wheezing” class was used as the reference group. PASW 23 (SPSS Inc., Chicago, IL, USA) was used to perform the analysis.

Wheezing data were available for 9353 Caucasian, singleton, term-born children, with the complete dataset of interest available for 6161 children. The number of children included in the adjusted analysis decreased due to missing data as only children with complete data were included. As expected (since preterm-born children were excluded), the gestational age and birthweight of included participants were higher than for the excluded children. Rates of SES, delivery by CS and IUGR were lower, but breastfeeding was higher, in included participants compared to excluded participants.

Table 1 reports unadjusted and adjusted associations between catch-up growth in the first year of life and wheezing phenotypes. Adjustments were made for important early-life factors associated with wheezing in later life including sex, IUGR, antenatal and postnatal smoking, SES, breastfeeding, childcare, postnatal smoking, and CS. Catch-up growth was associated with early wheeze but not persistent or late wheeze. When early-life factors were added into the model, children exposed to antenatal smoking, belonging to the lowest SES groups, or of male sex were all associated with all wheezing phenotypes. Children who were born with IUGR were associated with early wheeze but had a lower risk of developing late wheeze. Delivery by CS was associated with early and persistent wheeze but not late wheeze. Breastfeeding was associated with a slightly lower risk of early and persistent wheeze. Exposure to postnatal smoking was associated with early wheeze. Formal and informal childcare were associated with early and late wheeze. The association between catch-up growth and the early wheeze phenotype remained after adjustments for the above early-life factors. Sensitivity analyses using the weighted posterior probabilities of wheezing phenotypes resulted in essentially the same associations. Interestingly, there was a linear relationship between increase in weight gain z-scores and odds ratio of early wheeze ranging from 1.2 (95% CI 1.06–1.35) for increase of >0.4 weight gain z-score to 1.53 (1.22–1.93) for z-score of >2.0.

**TABLE 1:** Unadjusted and adjusted associations between weight gain in the first year of life and wheezing phenotypes using most-likely phenotype, and using the No/infrequent-wheezing phenotype as the reference category

	**Relative risk ratio (95% CI) p-value**		
	**Early wheeze**	**Persistent wheeze**	**Late wheeze**
**Unadjusted (n=9353)**			
Subjects n (%)	1232 (13.2%)	863 (9.2%)	295 (3.2%)
Weight gain 6–12 months of age^#^	1.29 (1.13–1.46) 8.40×10^−5^	1.12 (0.97–1.30) 0.13	1.01 (0.79–1.30) 0.93
**Adjusted**^¶^ **(n=6161)**			
Subjects n (%)	802 (13.0%)	581 (9.4%)	182 (3.0%)
Weight gain birth to 6–12 months of age^#^	1.21 (1.03–1.42) 0.02	0.99 (0.82–1.19) 0.89	1.04 (0.76–1.42) 0.82
Sex (Male)	1.25 (1.07–1.45) 0.01	1.81 (1.51–2.17) 8.66×10^-11^	1.15 (0.85–1.54) 0.37
IUGR	1.15 (0.91–1.45) 0.17	0.99 (0.74–1.32) 0.92	0.46 (0.24–0.88) 0.02
Smoking during pregnancy	1.37 (1.16–1.62) 0.00028	1.32 (1.09–1.61) 0.005	1.16 (0.83–1.62) 0.38
Social class (Lowest 5)	1.53 (1.24–1.90) 0.000091	1.12 (0.89–1.42) 0.343	1.55 (1.03–2.342) 0.04
Social class (4)	1.12 (0.79–1.58) 0.54	1.02 (0.70–1.49) 0.92	1.23 (0.64–2.38) 0.54
Social class (3)	1.05 (0.69–1.61) 0.82	0.72 (0.43–1.21) 0.22	0.15 (0.02–1.08) 0.06
Social class (2)	1.18 (0.94–1.48) 0.16	0.91 (0.70–1.19) 0.50	1.22 (0.78–1.89) 0.38
C-section delivery	1.10 (0.91–1.34) 0.31	1.27 (1.03–1.57) 0.03	0.81 (0.54–1.23) 0.33
Breastfeeding (Yes)	0.87 (0.73–1.03) 0.10	0.89 (0.72–1.07) 0.19	0.96 (0.69–1.33) 0.78
Postnatal smoking	1.11 (0.93–1.32) 0.27	0.96 (0.77–1.18) 0.67	0.99 (0.69–1.41) 0.95
Childcare (Formal)	1.35 (1.06–1.71) 0.01	0.71 (0.53–0.96) 0.02	1.27 (0.79–2.02) 0.33
Childcare (Informal)	1.09 (0.92–1.29) 0.34	0.89 (0.74–1.08) 0.24	1.22 (0.88–1.70) 0.23

Excluded: non-Caucasian, non-term, non-singletons. IUGR: intrauterine growth restriction. ^#^: catch-up growth was defined as a change in z-score of >0.67 between birth weight and weight at 6–12 months of age; ^¶^: adjusted for gender, IUGR, maternal smoking during pregnancy, social class, and c-section delivery using complete dataset.

These data show evidence that catch-up growth is associated with early wheeze, but not with persistent or late wheeze. As catch-up growth is generally promoted by clinicians for infants born with IUGR, it is important that the longer-term effect of catch-up growth is well defined and the risks and benefits clearly delineated. It seems that short-term catch-up growth could be detrimental leading to increased rates of early wheeze. This is possibly due to a lag in growth of the lungs, a process known as dysanapsis, whereby somatic growth exceeds that of lung growth. Moreover, there are potential effects of pro-inflammatory mediators released from excess adipose tissue (*e.g.* leptin) which may promote airway remodelling and hypersensitivity [14]. However, it is possible that in the longer term it may be beneficial as we previously reported that catch-up growth in term-born IUGR children, compared to children without catch-up growth, was associated with improvements in lung function [3]. In contrast, some evidence suggests that rapid early weight gain is not beneficial [4, 5]. We observed little or no evidence for catch-up growth associated with persistent and late wheeze possibly because different mechanisms such as atopy and insults, *e.g.* maternal smoking, may be more important. The associations between antenatal smoking and later respiratory outcomes are well recognised. Antenatal smoking can lead to abnormal fetal lung development as well as abnormal lung function and increased rates of asthma and wheezing in childhood [15]. Lung volumes increase more rapidly than airway calibre in early-life, but then proportionally less thereafter into early adolescence [16]. This may represent a regression of dysanaptic lung growth and thus a potential reduction in wheezing symptoms. More evidence from longitudinal studies to further investigate associations with wheezing phenotypes is required.

It would have been interesting to define IUGR using customised growth charts that take into account maternal factors associated with birthweight [17], as it is possible we may have categorised some children with IUGR using the LMS method who were genetically designed to be below the 10th centile and should have been in the control group. We may have categorised some children as being in the control group who were genetically designed to be born with a heavier birthweight and thus should have been assigned to the IUGR group.

A strength of this study is that we used data from a large, well-characterised UK cohort reporting well-defined wheezing phenotypes from the age of 3 years. Data were collected accurately at face-to-face interviews. The same associations were reported using weighted, most-likely assigned wheezing phenotype methods and after adjustment for early-life factors. Limitations are loss of follow-up, a lack of complete data for all subjects, and the lack of lung function data, which was not collected. There was possibly overlap between the exposure to the early-life factor and wheezing occurring. As wheeze was first reported at 3 years, we were unable to remove this overlap. The MCS oversampled from areas of deprivation and ethnic minority groups. We only reported data for Caucasian children and adjusted for SES to try to limit the influence these factors had.

In conclusion, we have reported the effect of catch-up growth in early-life and later wheezing phenotypes in term-born, singleton, Caucasian children. The results suggest that catch-up growth might have a detrimental effect on rates of early wheezing; thus, caution is required when instituting nutritional regimes to increase weight in infancy.

## Shareable PDF

10.1183/13993003.00884-2020.Shareable1 This one-page PDF can be shared freely online.
Shareable PDF ERJ-00884-2020.Shareable
