# Supplementary material for: The effect of catch-up growth in the first year of life on later wheezing phenotypes
Source: Eur Respir J. 2020 Dec 24;56(6):2000884. doi: 10.1183/13993003.00884-2020 (PMC7758542; doi:10.1183/13993003.00884-2020)
Supplement: Supplementary file 1 [file ERJ-00884-2020.Shareable.pdf]

AGORA
RESEARCH LETTER

SHAREABLE PDF

# The effect of catch-up growth in the first year of life on later wheezing phenotypes

Sarah J. Kotecha[1,3], John Lowe[1,3], Raquel Granell[2], W. John Watkins[1], A. John Henderson[2,†] and Sailesh Kotecha[1]

**Affiliations**: [1]Dept of Child Health, Cardiff University School of Medicine, Cardiff, UK. [2]MRC Integrative Epidemiology Unit, Population Health Sciences, Bristol Medical School, University of Bristol, Bristol, UK. [3]Joint first authors.

**Correspondence**: Sailesh Kotecha, Dept of Child Health, School of Medicine, Cardiff University, Heath Park, Cardiff, CF14 4XN, UK. E-mail: KotechaS@cardiff.ac.uk

@ERSpublications
**This study shows that catch-up growth in infancy is associated with increased early wheeze in childhood, so care is needed to not to excessively feed in early infancy** https://bit.ly/2YPxtBw

**Cite this article as:** Kotecha SJ, Lowe J, Granell R, *et al*. The effect of catch-up growth in the first year of life on later wheezing phenotypes. *Eur Respir J* 2020; 56: 2000884 [https://doi.org/10.1183/13993003.00884-2020].

This single-page version can be shared freely online.

*To the Editor*:

Although wheezing phenotypes have previously been accurately described using well-defined cohorts reporting longitudinal wheezing, early-life factors which lead to development of each wheezing phenotype remain uncertain [1, 2]. Birth weight and catch-up growth affect later respiratory outcomes [3], but the influence of weight gain on specific wheezing phenotypes in term-born children has not been described. Rapid weight gain in early-life is associated with increased rates of childhood wheeze and lower lung function [4, 5]. In one meta-analysis rapid infant weight gain was linked to pre-school wheeze and school-aged asthma; and to increased childhood respiratory symptoms in another meta-analysis [6, 7]. Effect of weight gain in early-life in term-born children on wheezing in early-life is less well reported [8]. In contrast, rapid increase in body mass index in infancy is associated with increased risk of asthma at school-age in preterm-born infants [9].

Copyright ©ERS 2020.. This version is distributed under the terms of the Creative Commons Attribution Licence 4.0.
